# Trastuzumab immunogenicity development in patients’ sera and in laboratory animals

**DOI:** 10.1186/s12865-021-00405-z

**Published:** 2021-02-19

**Authors:** Lobna Abdel Aziz Kilany, Ayman Abdel Samie Gaber, Mohammad Mabrouk Aboulwafa, Hamdallah Hafez Zedan

**Affiliations:** 1 National Organization for Research and Control of Biologicals (NORCB), 51 Wezaret El Zeraa St, El-Agouza-Giza, Dokki, Egyptian Drug Authority, P.O Box: 354, Dokki, Egypt; 2 Department of Oncology, National Cancer Institute, Cairo University, Kornish El-Nile - Fom El- Khaleg, Cairo, 11796 Egypt; 3 Department of Microbiology and Immunology, Faculty of Pharmacy, Ain Shams University, African union organization Street, Abbassia, Cairo, 11566 Egypt; 4 Faculty of Pharmacy, King Salman International University, Ras-Sedr, South Sinai Egypt; 5 Department of Microbiology and Immunology, Faculty of Pharmacy, Cairo University, Kasr El-Aini St, Cairo, 11562 Egypt

**Keywords:** Trastuzumab, Immunogenicity, ELISA, ACE assay, Lab animals

## Abstract

**Background:**

Immunogenicity is a major challenge in drug development and patient care. Clinicians and regulators are familiar with immunogenicity concerns of monoclonal antibody (mAb) therapeutics, growth factors and enzyme replacements. Although most small therapeutic molecules are unlikely to trigger undesirable immunogenic responses against themselves upon their administration, the biological therapeutic agents are likely to induce such kind of immunogenicity. This imparts a problem that has to be considered upon judging their risk–benefit ratio. In this article, we tested the immunogenicity developed in patients’ sera due to the use of trastuzumab and that developed in laboratory animals injected with this recombinant humanized IgG1 monoclonal antibody.

**Methods:**

We studied trastuzumab immunogenicity by: I in vitro detection of anti-trastuzumab antibody (Ab) levels in patient’s serum samples withdrawn at different points during trastuzumab treatment course; I.1 using an Affinity Capture Elution (ACE) assay, the assay is both sensitive and highly tolerant to free drug; I.2 using MTT cytotoxicity method against MCF-7 cell line as confirmatory method used in sample showed high level of anti-trastuzumab Ab and to determine neutralizing activity of the anti-trastuzumab Ab. II in vivo immunogenicity testing of trastuzumab in lab animals.

**Results:**

In vitro analysis of patients’ sera for antibodies developed against trastuzumab revealed that this monoclonal antibody has low immunogenicity since most samples showed low levels of anti-trastuzumab antibodies that decreased progressively along the treatment course. Only 1% of samples showed high levels of anti-trastuzumab antibodies which might affect treatment course. In vivo immunogenicity testing in mice showed also low immunogenicity of trastuzumab that could support the in vitro clinical assessment applied in our study.

**Conclusions:**

The study gives an evidence for the low trastuzumab immunogenicity when assessed in Egyptian patients under treatment with this biological therapeutic agent. This supports its prescription and continuous use across the approved indications as biological therapeutic agent.

## Background

Immunogenicity is the undesired immune response due to the development of an adaptive immune response to a therapeutic agent [1–3]. Typically, this reaction occurs when foreignness or stress signals are perceived by the immune system, which triggers the humoral response by developing specific anti-drug antibodies (ADAs; also referred to as anti-therapeutic antibodies (ATAs)) to the therapeutic agent. Clinicians and regulators can’t predict the clinical relevance of these ADAs as has ranged from no clinical relevance detected up to life-threatening responses [3].

A study for evaluation and reporting immunogenicity data, review the prescribing information and clinical review of Food and Drug Administration (FDA) for 121 approved biological products show that 89% of the products had reported immunogenic (IG), and 49% of the immunogenic products showed impact on its efficacy [4]. Biotherapeutic proteins are used to treat a wide variety of diseases, ranging from the rare (e.g., hemophilia) to the common (e.g., diabetes and cancer). An important safety and efficacy concern with biotherapeutic proteins is the risk of immunogenicity. For some biotherapeutics, the prevalence of ADAs in the patient population can be as high as 87% [5]. ADAs sometimes affect the biological function of the biotherapeutic, and these are called neutralizing ADAs (nADAs) or neutralizing antibodies (NABs). However, even antibodies that do not directly affect the function of the proteins (sometimes referred to as binding antibodies) can enhance, or impede, the clearance of the therapeutic agent, and thus affect a drug’s pharmacokinetics (PK) and/or the pharmacodynamics (PD). Risks of immunogenicity can range from no clinical adverse effects to life threating adverse effects as ADA can impact both safety and bioavailability of the drug. In rare cases, immune responses can be severe, resulting in hypersensitivity reactions and even death [6]. The most common adverse reactions associated with trastuzumab products in the metastatic breast cancer setting are fever, nausea, vomiting, infusion reactions, diarrhea, infections, increased cough, headache, fatigue, dyspnea, rash, neutropenia, anemia, and myalgia [7]. In clinics, safety measures should be taken in case of immunogenic response detected; immunogenicity may require dose escalations, alternative treatments, frequent testing, and hospitalizations [1, 5]. Anticancer chemotherapy involving trastuzumab should be administered only to patients for whom treatment with Herceptin is judged to be appropriate, at medical institutions where effective emergency treatment can be provided. The immunogenicity of many clinically approved products has been documented very well while some have failed even before reaching clinical trials. The development of ADA can affect efficacy and safety of the therapeutic proteins [8]. Different factors may influence the immunogenicity of therapeutic proteins: structural features (variation of sequence and glycosylation), change in labelled storage conditions (cause denaturation or aggregation), if preparation contains any contaminants or impurities, duration and frequency of treatment, and route of administration; all of these factors or relevant factors elicit complex interactions between the patient’s body and the biological drug [9]. Non-clinical immunogenicity assessments are very useful. They can be used to select candidate drugs in early stages of development [8, 10]. In our study we used In vivo immunogenicity testing of trastuzumab in lab animals and the data obtained supported results of the in vitro assay for trastuzumab immunogenicity in patient’s sera. There are two types of ADA formed, either persistent or transient, the latter type (transient ADA) is of less concern as it found rarely impact clinical outcomes and cause adverse events. Formation of ADA may occur immediately after initiation of mAb treatment course or later during treatment when reaching the concentrations that completely neutralize the drug. All regulators requirements include an accurate and reliable detection of ADA to mAb in clinical trials for ensuring safety of the drug and aids in understanding efficacy of mAb [11]. Trastuzumab (Herceptin®; Genentech, San Francisco, CA, USA) is a recombinant humanized monoclonal antibody IgG1 targeting human epidermal growth factor receptor-2 (HER2) and has substantially improved the prognosis of HER2-positive breast cancer [12]. Administration of the intravenous (IV) formulation of trastuzumab that we used in our study can be either weekly infusions (initial dose of 4 mg/kg followed by subsequent doses of 2 mg/kg) or every 3 weeks (initial dose of 8 mg/kg followed by subsequent doses of 6 mg/kg) according to the label recommendation and depending on the indication the regimen chosen. For new drug approvals immunogenicity detection is required in clinical trials and can be detected by measurement of ADA in patients. Different assays can be used for immunogenicity detection of mAb, assays must be sensitive enough to detect low levels of ADA and be able to differentiate between the drug and the ADA, selecting an appropriate method is very important to reduce false results and clearly detect immunogenicity rates and severity [13–18]. Trastuzumab as a monoclonal antibody could induce the activation of a humoral immune response generating anti-drug antibodies (ADAs) which could block the action of trastuzumab and forming immune complexes which decrease its efficacy. Many studies measured trastuzumab immunogenicity, Hanna studied trastuzumab immunogenicity using highly sensitive assay and compared between different routes of administration (SC/IV formulation). ADA against both formulas of trastuzumab were detected transiently and were of no relevance in terms of efficacy and safety [18–22]. Clinical studies were conducted and immunogenicity of IV trastuzumab with biosimilar drugs was compared. The results reported that both trastuzumab and its biosimilar drugs showed markedly low ADA, that indicate low immunogenicity of trastuzumab. Furthermore, none of the patients included in the studies and who were ADA positive presented significant adverse events (AEs) related to immunogenicity or show a significant difference in efficacy and safety results [23–27]. The present study focused on in vivo immunogenicity testing of trastuzumab in lab animals and in vitro detection of anti-trastuzumab antibody (Ab) levels in Egyptian patients’ serum samples withdrawn at different points during trastuzumab treatment course. The in vivo and in vitro analysis of trastuzumab immunogenicity revealed that it’s of low immunogenicity among Egyptian patients and supports data for Herceptin (trastuzumab) which show that trastuzumab has low immunogenicity across all indications and its clinical continuous use as biologic therapeutic agent [7, 9, 19, 26, 28].

## Results

Table 1 shows a summary of immunogenicity testing that was conducted in laboratory animals and patients’ sera and results obtained.

**Table 1:** Summary of immunogenicity testing of trastuzumab in laboratory animals and patients’ sera

Applied approach	(A) In vivo immunogenicity testing of trastuzumab in lab animals		
	(B) In vitro detection of anti-trastuzumab Ab levels in patient’s serum samples		
Methodology	Approach A	**Using one control group and 3 groups injected with different concentrations of trastuzumab.**	
	Approach B	**Using ACE assay (analysis of serum samples of 101 patients)**	
		1	Analysis of 18 patient’s serum samples withdrawn before trastuzumab treatment course
		2	Analysis of 46 patient’s serum samples withdrawn at a single point during trastuzumab treatment course
		3	Analysis of serum samples of 32 patients withdrawn at 2 different points during trastuzumab treatment course
		4	Analysis of 5 patient’s serum samples withdrawn after trastuzumab treatment course
		**Using MTT cytotoxicity assay**	
			Determination of neutralizing activity of anti-trastuzumab Ab in a single patient’s serum sample that showed highest ADA titer
Results	Approach A	Results are presented in Fig. 1 - Trastuzumab showed low immunogenicity in mice since the anti-trastuzumab Ab levels developed in tested groups injected with the drug didn’t exceed 1.3	
	Approach B	**Using ACE assay (analysis of serum samples of 101 patients)**	
		1	- Only 3 serum samples showed anti-trastuzumab Ab titers between 0.7–0.8 while the rest samples showed no detected anti-trastuzumab Ab
		2	Results are presented in Table 2 - 30 samples showed no detected anti-trastuzumab Ab while the remaining 16 samples showed variable levels of anti-trastuzumab Ab titers ranging from 1.07–8.18.
		3	Results are presented in Table 3 - at the 1st assay point 16 out of 32 tested samples showed variable levels of anti-trastuzumab Ab ranging from 1.13–4.64, while at the 2nd assay point only 9 out of 32 tested samples showed variable levels of anti-trastuzumab Ab ranging from 1.01–2.4
		4	Results are presented in Table 4 -Only two serum samples coded T3–99 and T3–100 of two patients showed elevated levels of ADAs of 103.63 and 7.13, respectively. The patient of the serum sample coded T3–99 didn’t complete the 17 doses of trastuzumab treatment course as she didn’t respond clinically to treatment. She only received 2 doses of trastuzumab
		**Using MTT cytotoxicity assay**	
			Results are presented in Table 5 - the neutralization of trastuzumab activity was observed when: 1- the drug and serum preparations were added simultaneously to MCF-7 cell line 2- the serum preparations were added to MCF-7 cell line one hour before drug addition

### In vivo immunogenicity testing of trastuzumab in lab animals

**Fig. 1:**
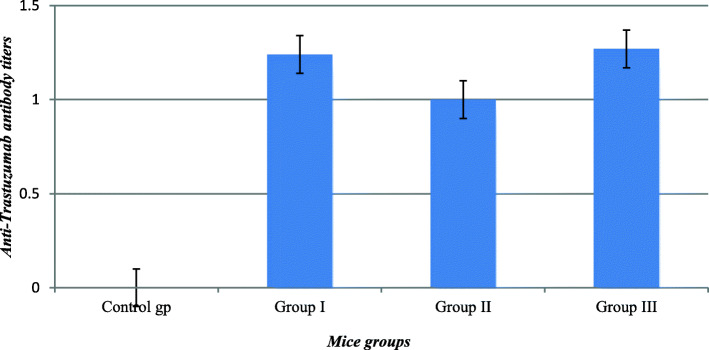
Anti-trastuzumab antibody levels developed in mice injected with different concentrations of trastuzumab. Control group, mice injected with only normal saline; group I, mice injected with 14.8 mg trastuzumab/kg body weight; group II, mice injected with 74 mg trastuzumab/kg body weight; group III, mice injected with 148 mg trastuzumab/kg body weight

Male Swiss albino mice were used for in vivo immunogenicity testing of trastuzumab using four groups of lab animals, each consisting of seven mice. At the end of the experiment, the remaining survived mice were five in case of control group, three in group I, three in group II and two in group III. Figure 1 shows the antibody titers developed in the pooled serum of each group. The results obtained revealed that the trastuzumab is of low immunogenicity in mice since the anti-trastuzumab Ab levels developed in tested groups injected with the drug didn’t exceed 1.3. The death in lab animals injected with trastuzumab could be attributed to the drug cardiotoxicity effect as evidenced by the occurrence of death in all animals injected with high concentration of the drug (370 mg of trastuzumab/kg body weight). The concentration used was five folds higher than human equivalence dose. This guided us to lower the dose used in our experiments.

### In vitro detection of anti-trastuzumab antibody levels in patient’s serum samples

#### Using ACE assay

##### Before Trastuzumab treatment course

Any anti-trastuzumab Ab levels developed in patient’s serum before starting the trastuzumab treatment course was determined. The results of serum samples from 18 patients subjected to analysis revealed that only 3 serum samples showed anti-trastuzumab Ab titers between 0.7–0.8 while the rest samples showed no detected anti-trastuzumab Ab. However in this study these low levels (< 1) of anti-trastuzumab Ab titers were considered as artifacts.

##### At a single point during Trastuzumab treatment course

The anti-trastuzumab Ab levels developed in serum samples of 46 patients were determined within the trastuzumab treatment course and samples were divided according to the number of doses administered before blood sample withdrawal. The results of tested serum samples are shown in Table 2. The results revealed that 30 samples showed no detected anti-trastuzumab Ab while the remaining 16 samples showed variable levels of anti-trastuzumab Ab titers ranging from 1.07–8.18.

**Table 2:** Anti-trastuzumab Ab levels developed in 46 patien’s serum samples and determined at a single point during trastuzumab treatment course

Administered doses before blood sample withdrawal	Sample Code	Titer		*Percentage of positive samples
		Value	±SD	
1	T1–19	ND		33.33%
	T1–20	4.61	0.62	
	T1–21	0.49	0.04	
	T1–22	ND		
	T1–23	3.64	0.02	
	T1–24	ND		
2	T1–25	0.73	0.04	66.67%
	T1–26	2.86	0.04	
	T1–27	6.65	1.78	
3	T1–28	ND		75%
	T1–29	1.27	0.02	
	T1–30	2.52	0.05	
	T1–31	5.31	0.07	
4	T1–32	0.89	0.04	50%
	T1–33	ND		
	T1–34	7.78	0.24	
	T1–35	8.18	0.60	
5	T1–36	0.56	0.03	NVC
6	T1–37	ND		
7	T1–38	ND		
	T1–39	1.07	0.07	
8	T1–40	ND		33.33%
	T1–41	ND		
	T1–42	ND		
	T1–43	2.03	0.1	
	T1–44	2.77	0.44	
	T1–45	ND		
10	T1–46	ND		25%
	T1–47	ND		
	T1–48	ND		
	T1–49	2.9	0.001	
12	T1–50	ND		NVC
	T1–51	ND		
13	T1–52	ND		
	T1–53	ND		
14	T1–54	3.36	0.06	16.67%
	T1–55	ND		
	T1–56	ND		
	T1–57	0.98	0.08	
	T1–58	ND		
	T1–59	ND		
15	T1–60	2.14	0.07	NVC
	T1–61	0.99	0.03	
16	T1–62	ND		
	T1–63	ND		
17	T1–64	5.77	0.27	

*Percentage of positive samples was calculated from samples that gave titers ≥1 relative to the number of tested samples of patients administered the same number of doses

*ND* no detected antibodies, *NVC* not valid for calculations

**Fig. 2:**
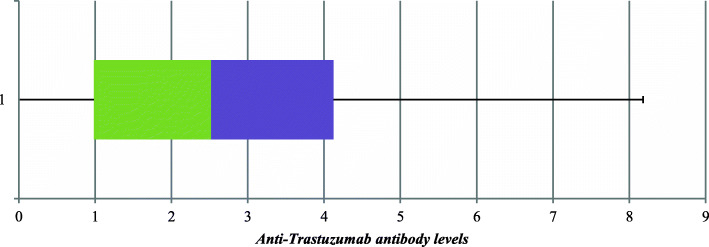
Box and whisker diagram for anti-trastuzumab Ab levels developed in 46 patient’s serum samples and determined at a single point during trastuzumab treatment course

The results of serum samples from the 46 patients shown in Table 2 were further represented using box-and-whisker diagram (Fig. 2). In such plot method, data entry has to be divided into five quartiles; minimum, lower, median, upper and maximum value. In the plot, one box is constructed extending from the lower quartile to the upper one. The box length represents the difference between the two quartiles, the upper and the lower ones. The median of the total dataset appears in the middle of the box as vertical line. In both directions of the boxplot, two lines can be drawn extending outside the box to give two additional vertical lines, one vertical line close to the upper quartile (termed upper whisker) and another one close to the lower quartile (termed lower whisker). This diagram shows that the median of all results was calculated to be 2.52 with upper quartile 4.12 and lower quartile 0.99 within the box. The interquartile range from the box was extended to represent minimum value 0 (serum samples showing no detectable anti-trastuzumab Ab) and maximum value of 8.18 (the largest value determined in serum samples)

##### At 2 different points during Trastuzumab treatment course

The anti-trastuzumab Ab levels developed in serum samples of 32 patients were determined at 2 different points during trastuzumab treatment course and the results are shown in Table 3. The results revealed that at the 1st assay point 16 out of 32 tested samples showed variable levels of anti-trastuzumab Ab ranging from 1.13–4.64, while at the 2nd assay point only 9 out of 32 tested samples showed variable levels of anti-trastuzumab Ab ranging from 1.01–2.4.

**Table 3:** Anti-trastuzumab Ab levels developed in 32 patien’s serum samples and determined at two different points during trastuzumab treatment course

Sample code	No. of administered doses before 1st assay point	1st assay point titer			No. of administered doses before 2nd assay point	2nd assay point titer		
		Value	±SD	*Percentage of positive samples		Value	±SD	**Percentage of positive samples
T2–65	1	1.36	0.01	71.43%	12	ND		28.13%
T2–66		1.94	0.02		10	1.65	0.04	
					12	ND		
T2–67		ND			10	ND		
T2–68		1.61	0.02		16	ND		
T2–69		ND			10	ND		
T2–70		3.1	0.01		7	ND		
T2–71		2.77	0.05		10	1.37	0.07	
T2–72	2	ND		80%	12	ND		
T2–73		1.78	0.06		14	2.25	0.01	
T2–74		1.56	0.09		12	1.02	0.03	
T2–75		3.07	0.05		14	2.4	0.04	
T2–76		2.93	0.1		10	1.01	0.04	
T2–77	3	ND		0%	8	ND		
					15	ND		
T2–78		ND			17	ND		
T2–79		ND			15	ND		
T2–80	4	ND		33.33%	6	ND		
T2–81		2.24	0.02		14	ND		
T2–82		0.85	0.1		12	ND		
T2–83		ND			16	ND		
T2–84		ND			14	ND		
T2–85		1.13	0.01		14	1.33	0.001	
T2–86	6	ND		25%	13	ND		
T2–87		ND			15	ND		
T2–88		2.43	0.01		13	ND		
T2–89		ND			14	ND		
T2–90	7	2.63	0.16	67%	13	1.18	0.03	
T2–91		ND			16	ND		
T2–92		4.64	0.03		17	1.67	0.05	
T2–93	8	ND		50%	17	ND		
T2–94		0.85	0.01		15	ND		
T2–95		1.29	0.08		16	ND		
T2–96		1.95	0.06		16	ND		

*Percentage of positive samples was calculated from samples that gave titers ≥1 relative to the number of tested samples of patients administered the same number of doses

**Percentage of positive samples was calculated from samples that gave titers ≥1 relative to the total number of samples in 2nd assay point

**Fig. 3:**
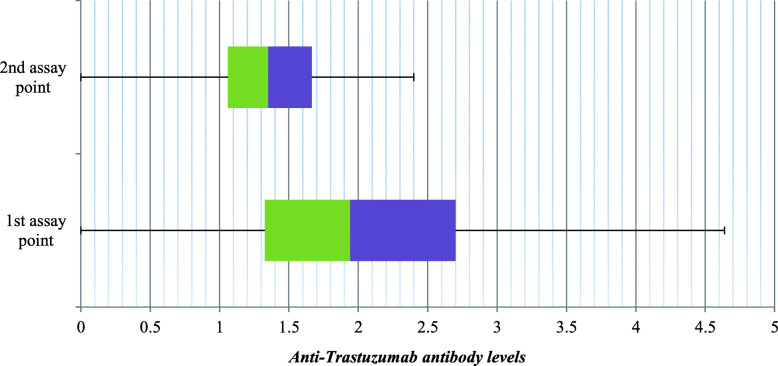
Box and whisker diagram for anti-trastuzumab Ab levels developed in serum samples of 32 patients and determined at two different points during Trastuzumab treatment course

The result of serum samples from the 32 patients shown in Table 3 were further represented using box-and-whisker diagram (Fig. 3). This diagram shows that in the 1st assay point the median of all results was calculated to be 1.94 with upper quartile 2.7and lower quartile 1.33 within the box. The interquartile range from the box was extended to represent minimum value 0 (serum samples showing no detectable anti-trastuzumab Ab) and maximum value of 4.64 (the largest value determined in serum samples). While in the 2nd assay point the diagram shows that the median of all results was calculated to be 1.35 with upper quartile 1.67 and lower quartile 1.06 within the box. The interquartile range from the box was extended to represent minimum value 0 (serum samples showing no detectable anti-trastuzumab Ab) and maximum value of 2.4 (the largest value determined in serum samples).

##### After trastuzumab treatment course

The anti-trastuzumab Ab levels developed in patient’s serum samples were determined after the 17 doses of trastuzumab treatment course (the standard treatment schedule is 1 dose every 3 weeks, however there was a deviation from this schedule and the treatment course was completed over a period 12–18 months). The results of serum samples from the 5 patients subjected to analysis are shown in Table 4. However, the patient of the serum sample coded T3–99 didn’t complete the 17 doses of trastuzumab treatment course as she didn’t respond clinically to treatment. She only received 2 doses of trastuzumab.

**Table 4:** Anti-trastuzumab Ab levels developed in patien’s serum samples after trastuzumab treatment course

Sample code	Titer		*Elapsed time (months)
	Value	±SD	
T3–97	ND		4
T3–98	ND		11
T3–99	103.63	5.58	22
T3–100	7.13	0.1395	9
T3–101	ND		7

*Elapsed time: time passed after last dose of treatment course till sample withdrawal

**Fig. 4:**
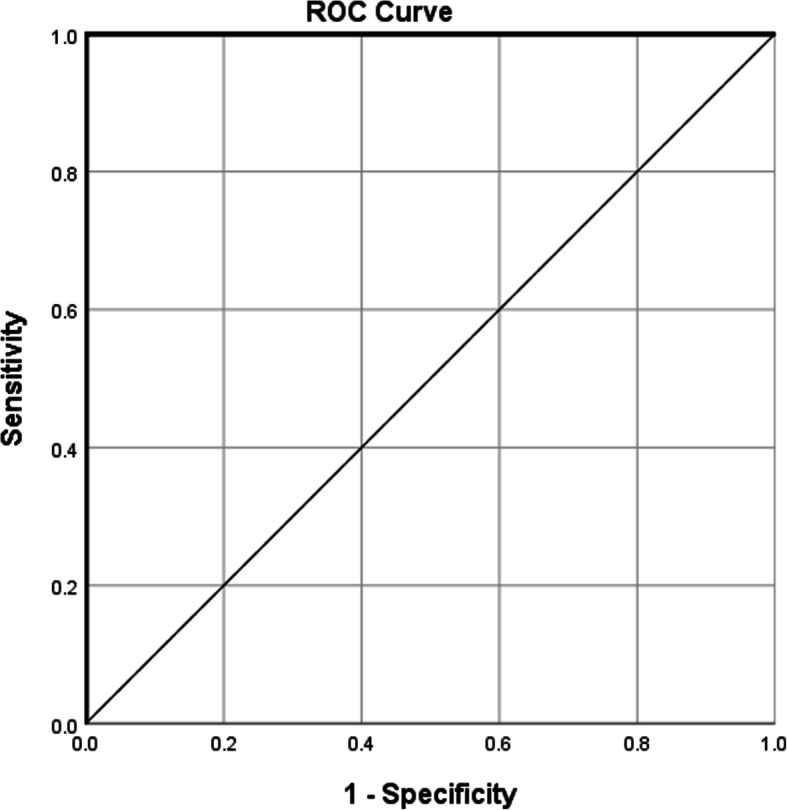
ROC curve for ACE assay analysis results. The area under the curve (AUC) represents the test result variable: ADA titer values from ACE assay of all serum samples, larger values of the test result variable indicate stronger evidence for a positive actual state

ACE assay validation parameters were calculated using IBM SPSS Statistics Data Editor. To determine assay cutoff point (the level of response above which a sample is defined as positive), all ADA titers are represented in a ROC curve as shown in Fig. 4. From sensitivity and specificity of the titer data shown in Fig. 4, the calculated cutoff point was found to be 1.

#### Using MTT cytotoxicity assay

**Fig. 5:**
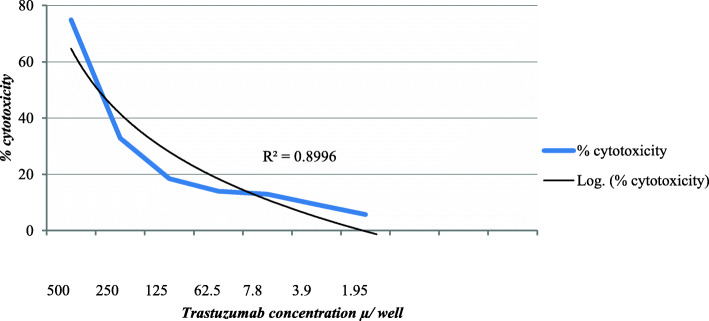
Cytotoxicity profile of trastuzumab against MCF-7 cell line as determined by MTT cytotoxicity assay

MTT cytotoxicity assay was used to determine neutralizing activity of anti-trastuzumab Ab in single patient serum sample coded T3–99 which showed the highest Ab titer by ACE assay. The cytotoxicity of trastuzumab (Herceptin) on MCF-7 was determined first. Figure 5 shows the cytotoxicity profile of trastuzumab on the tested cells from which it can be demonstrated that 250 μg/well trastuzumab produced about 30% cytotoxicity. The 250 μg/well trastuzumab dose was mixed with three dilutions of sample patient serum T3–99 in presence of MCF-7 cells using three different regimens as described in materials and methods. Results of cytotoxicity assay in Table 5 showed that the neutralization of trastuzumab activity could be observed in regimen 1 (the drug and serum preparations were added simultaneously to MCF-7 cell line) and regimen 2 (the serum preparations were added to MCF-7 cell line one hour before drug addition).

**Table 5:** MTT cytotoxicity assay of neutralizing activity of anti-trastuzumab Ab developed in patient’s serum using MCF-7 cell line

Serum dilutions	% Cytotoxicity							
	Control		1st Regimen		2nd Regimen		3rd Regimen	
	Value	±SD	Value	±SD	Value	±SD	Value	±SD
**0**	36.89	0.08	36.89	0.08	36.89	0.08	36.89	0.08
**1:20**			49.19	0.12	29.88	0.05	34.19	0.04
**1:100**			12.12	0.27	1.49	0.12	25.98	0.10
**1:200**			0.76	0.15	13.71	0.02	50.22	0.08

**Control:** Drug alone added to MCF-7 cell line

**1st Regimen:** Both drug and serum dilutions were added simultaneously to MCF-7 cell line

**2nd Regimen:** The serum dilutions were added to MCF-7 cell line 1 h before addition of the drug

**3rd Regimen:** Both drug and serum dilutions were mixed at equal volumes and left at room temperature for 1 h before their addition to MCF-7 cell line

**Fig. 6:**
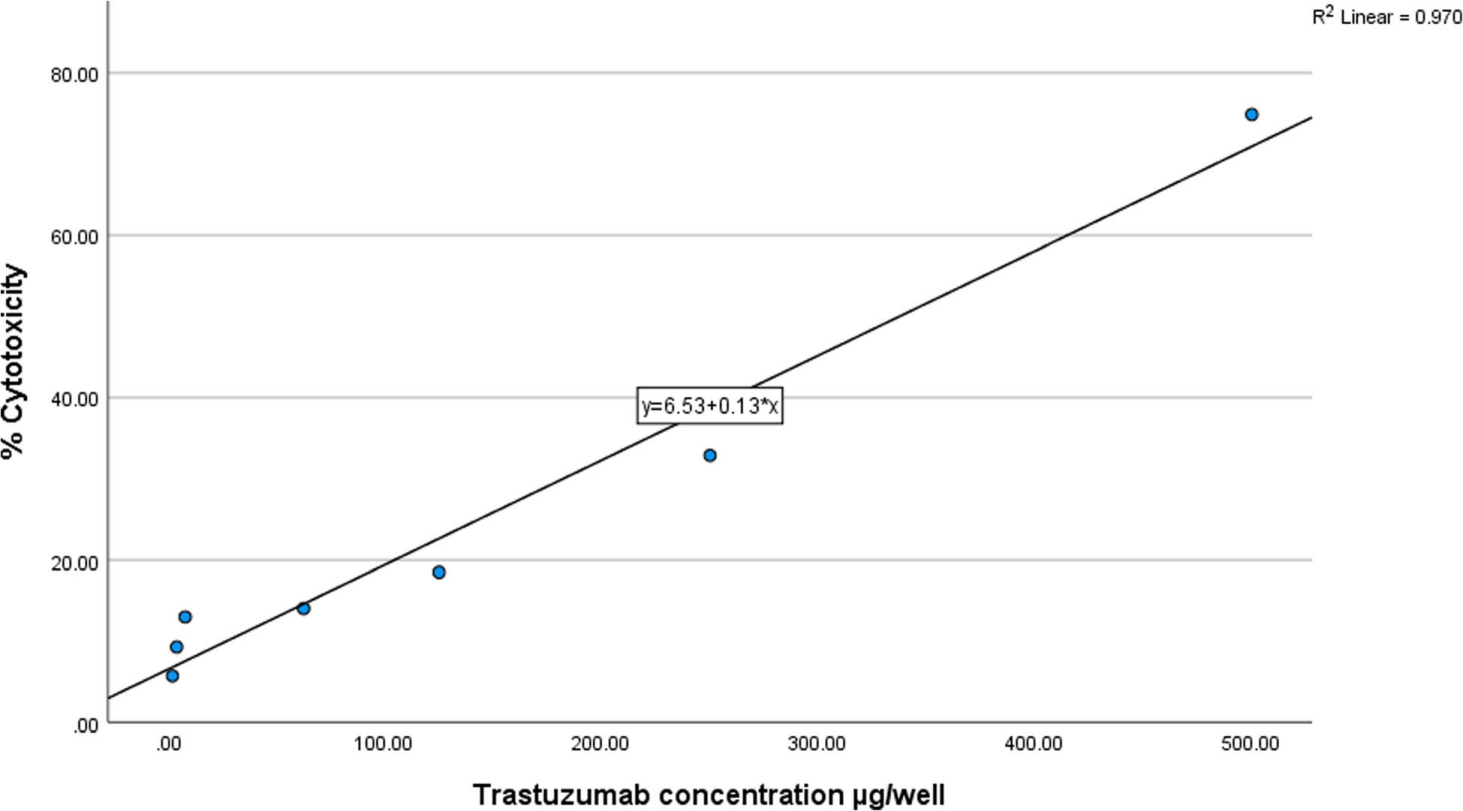
Scatter plot of cytotoxicity (expressed as percentage) caused by various concentrations of trastuzumab against MCF-7 cell line

IBM SPSS Statistics Data Editor was used to validate the results obtained by MTT cytotoxicity assay. The cytotoxicity profile of trastuzumab against MCF-7 cells was presented as a scatter plot shown in Fig. 6. The figure shows the correlations with R^2^ of 0.97 and regressions with the equation shown below.

$$ \mathrm{Y}=6.53+0.13\ast \mathrm{X} $$

Where Y is the cytotoxicity percentage and X is trastuzumab concentration (μg/well)

## Discussion

Trastuzumab therapeutic monoclonal Ab is dosed at high levels and has relatively long half-life resulting in significant blood levels for extended periods of time. The presence of high concentration of circulating drug could make detection of anti-trastuzumab Ab very difficult, so in the present study blood samples collection was done after washouts of the drug and in vitro detection of anti-trastuzumab antibody levels in patient’s serum samples was performed using ACE assay method. ACE assay method is sensitive enough to detect low levels of ADA as well as it involves the dissociation of ADA-drug complexes with acid treatment, this enable determination of total ADA developed in patient’s serum.

In vitro detection of anti-trastuzumab Ab levels in serum samples of 18 patients before trastuzumab treatment course showed no detectable anti-trastuzumab Ab in all patients except in three samples which showed low level (< 1) of anti-trastuzumab Ab titers. These ADA may be due to non-specific reactions during the assay process and/or non-recognized pre exposure to the drug or other relevant drugs that induced cross reactions with the target drug. In vitro detection of anti-trastuzumab Ab levels in serum samples of 46 patients at a single point during trastuzumab treatment course revealed that 16 (34.8%) samples gave variable levels of anti-trastuzumab Ab titers ranging from 1.07–8.18. Comparing the results of tested serum samples at various administered doses (1, 2, 3, 4, 8, 10 and 14, those represented by ≥ three serum samples), the results showed that there is a relationship between the number of administered doses and the percentage of positive samples up to 3 administered doses. Higher administered doses showed a dose dependent reduction in percentage of positive samples. This could be due to the tolerance developed by the body after the initial administered doses. In vitro detection of anti-trastuzumab Ab levels in serum samples of 32 patients at 2 different assay points during trastuzumab treatment course showed that in the 1st assay point 50% of samples showed variable levels of anti-trastuzumab Ab ranging from 1.13–4.64 (Table 3). While in the 2nd assay point the percentage decreased to be only 28.13% of samples that showed variable levels of anti-trastuzumab Ab ranging from 1.01–2.4. From the results it appears that the percentages of patients showed anti-trastuzumab Ab obviously decreased in the 2nd assay point and also the maximum value determined in the tested samples was lower than that obtained in the 1st assay point (2.4 in the 2nd assay point versus 4.64 in the 1st assay point). Additionally, most samples showed either marked decrease or no detectable Ab levels when they assessed in the 2nd assay point (Table 3). In case of trastuzumab, ADA developed in most patients seems to be transient as they decreased progressively along the treatment course. This observation was supported by the clinical outcomes recorded for the patients under study since they showed positive response to the treatment with the drug and no adverse events were detected during trastuzumab treatment course. The results obtained in our study are in agreement with those reported by Bian et al. [11], who stated that ADA can be either persistent or transient, of which the latter kind is of less concern as transient ADA rarely impact clinical outcomes and adverse events. Immunogenicity detected after complete trastuzumab treatment course revealed that only 2 patient’s serum samples gave significant levels of anti-trastuzumab Ab (Table 4). The serum sample coded T3–99 (withdrawn 22 months after last trastuzumab dose) and T3–100 (withdrawn 9 months after last trastuzumab dose) showed persistent levels of anti-trastuzumab Ab. However serum sample T3–100 was of lower levels of anti-trastuzumab Ab titer (7.13) compared to the anti-trastuzumab Ab level obtained in case of serum sample T3–99 (103.33). Clinically, the patient of sample coded T3–100 could complete 17 doses of trastuzumab treatment course without negative impact in clinical outcome. On the other hand patient with serum sample coded T3–99 experienced negative clinical outcomes that ended by the stop of trastuzumab administration after the 2nd dose. It can be summed up that among 101 patients, serum analysis for detection of anti-trastuzumab Ab, only one patient (1%) showed high level of persistent anti-trastuzumab Ab. The clinical unresponsiveness of that patient (serum sample T3–99) could be attributed to the development of high titer of anti-trastuzumab Ab as detected by analysis of her serum (Table 4). Trastuzumab as humanized mAb (produced by replacing most mouse sequence derived amino acids for human sequences, only the complementarity determining regions (CDRs) of the variable (v) regions of mouse sequence origin remain) has less ability to trigger immunogenic responses compared with murine (purified derived mouse Ab) and chimeric Ab (replacing murine constant regions with human constant regions). As a result, in humanized Ab T cell epitopes were so much reduced and found only in CDRs. Consequently, a likely reduction of the immunogenic potential while retaining full biologic function. This is the way by which immunogenicity of insulin was much reduced by the development human insulin [29, 30]. This may explain the observed low immunogenicity in this and other studies. However, some humanized and even fully human mAb still carry immunological risks [30, 31].

MTT cytotoxicity assay was used as confirmatory method for the in vitro detection of anti-trastuzumab Ab levels in patient’s serum samples and to determine neutralizing activity of anti-trastuzumab Ab using a single patient serum sample (sample coded T3–99). MCF-7 cell line could respond to a higher concentration of trastuzumab as 250 μg that produced about 30% cytotoxicity, this enabled us to detect the neutralizing activity of the developed Ab. The usage of 3 regimens of loading drug and serum on MCF-7 cell line showed that the neutralization of trastuzumab activity could be observed in regimen 1 (the drug and serum preparations added simultaneously to MCF-7 cell line) and regimen 2 (the serum preparations were added to MCF-7 cell line 1 h before drug addiction). On the other hand this activity wasn’t demonstrated in case of regimen 3 (both drug and serum preparations were mixed and left at room temperature for 1 h before their addition to MCF-7 cell line). This observation indicates that the mechanism of detected neutralizing activity of anti-trastuzumab Ab may be due to their effect on cell line rather than on the drug itself. It’s known that the degree of immunocomplex formation depends on the relative concentrations of Ab and drug molecules. As a result interaction between the Ab and the cell line depends on its degree of association/dissociation from its immunocomplex. Aize Kijlstra et al., reported that the immunocomplex dissociates by dilutions [32].

In vivo immunogenicity testing of trastuzumab in lab animals revealed absence of relationships between drug concentration, Ab titer, treatment period and the number of doses administered in each group of tested animals. However the low immunogenicity of trastuzumab detected in lab animals was comparable to the anti-trastuzumab Ab levels measured in clinical serum samples. This gives an evidence of low immunogenicity of trastuzumab that could support its continuous use as biological therapeutic agent.

## Conclusion

In this study trastuzumab showed results that indicate its low immunogenicity as biological therapeutic agent in pre-clinical and clinical studies. As very low percentage of patients can show high titer of anti-trastuzumab antibodies that is impacted clinically, it is much recommended to take into consideration detection of immunogenicity of trastuzumab in patients showing any serious adverse events or unresponsiveness to treatment after starting trastuzumab treatment course.

## Methods

The present study was approved by The Institution Review Board (IRB) of National Cancer Institute (NCI) under the IRB No. IRB00004025. This approval was issued according to ICH-GCP guidelines. Signed informed consents were obtained from all patients whom their blood samples and clinical data were used in this study. Additionally, another approval by the Safety and Occupational Health Committee at Faculty of Pharmacy, Cairo University, Cairo, Egypt with serial No. of MI (1481) has certify that this study doesn’t affect the health and safety.

All methods involving human participants, human sera and human data were carried out in accordance with Declaration of Helsinki and approved by IRB of NCI. All methods involving animal use were conducted in accordance with Research Ethics Committee (REC) for experimental and clinical studies at Faculty of Pharmacy, Cairo University, Cairo, Egypt which approved the conducted research. All experimental protocols were approved by Faculty of pharmacy, Cairo University, Cairo, Egypt.

### Animals

Male Swiss albino mice (Vacsera animal house, Egypt.) were used in the in vivo immunogenicity study.

### Blood samples and sera

Blood samples from 101 patients diagnosed with breast cancer (stages I-IV as classified and categorized according to TNM [tumor, node, and metastasis] stage classification) were obtained from NCI, Cairo, Egypt. In some cases more than one blood sample were obtained from the same patient. The blood samples were provided in Serum Separating Tubes (SST, KEMICO vacutainer, Z serum Clot Activator with Gel) [33]. Sera were prepared from all collected blood samples by allowing the blood containing tubes (approximately 5 ml aliquots) to clot for a minimum of 60 min in a vertical position at room temperature following by centrifugation at 3000×g for 10 min. The supernatants were separately aliquoted into 2–3 portions and stored at − 40 °C until use. Blood samples were collected from patients who their tumor biopsies were HER2 positive and under treatment with trastuzumab. Clinical data for all patients included in the study were reviewed and any AEs was recorded. Nearly all patients (100 out of 101) showed no observed AEs during the treatment course except infusion related reactions (IRRs) like fever, redness and swelling that were observed in few patients, and it was not known if these reactions related to trastuzumab or any other combined drugs. Only one female out of 101 included patients, her clinical data showed unresponsiveness for treatment with trastuzumab and the treatment was stopped after the 2nd dose.

### Cell line and its maintenance

Human breast cancer cell line MCF-7 (ATCC® HTB-22™) was obtained from Vacsera, Cairo, Egypt. The cell line was routinely cultured in 75-ml tissue culture flasks (Nunc Thermofisher Scientific) in RPMI 1640 medium supplemented with 10% heat inactivated fetal bovine serum, 2 mM non-essential amino acids, 2 mM sodium pyruvate, 100 units/ml penicillin and 100 μg/ml streptomycin (all from Sigma, USA), the cultured cells were incubated in CO_2_ incubator at 37 °C and 5% CO2.

### Chemicals and reagents

The chemicals used in the present study included: NaCO_3_ and NaCl (El Nasr pharmaceutical chemicals Co., Egypt), NaHCO_3_ (Biowest Co., USA), Tris/Trizma base and Tween 80 (Sigma, USA), Orthophosphoric acid 85% (Sigma-Aldrich, GmbH, Germany) and Acetic Acid, glacial extra pure (Sham lab, Syria). The reagents used in the present study included: BupH carbonate–bicarbonate buffer, 0.01 M Tris-Buffered Saline (TBS), TBST (TBS containing 0.05% Tween 80). Therapeutic monoclonal antibody trastuzumab (Tmab) was supplied by Roche Diagnostics (GmbH, Germany), Streptavidin poly -HRP was obtained from Pierce (Pierce Biotechnology, Rockford, IL, N200) and Biotin-Trastuzumab Conjugate (B-Tmab) was prepared by labeling of therapeutic humanized monoclonal antibody Trastuzumab (Tmab) using EZ-Link Sulfo-NHS-LC-Biotinylation Kits (Pierce, 21,435) according to manufacturer’s instructions.

### In vivo immunogenicity testing of trastuzumab in lab animals

Four groups (one control group and three groups for different concentrations of trastuzumab 14.8, 74 and 148 mg/kg) each contain 7 mice were assigned. Animals were 6–7 weeks old and weighed between 18 and 22 g at the initiation of dosing.

### Dose preparation and administration for animal experiments

Three dose levels of 14.8, 74 and 148 mg/kg [the middle dose of 74 mg/kg were chosen depending on mouse equivalence dose to human maintenance dose of 6 mg/kg. The animal equivalent dose (AED) was calculated from data adapted from FDA draft guidelines with slight modifications. Thereafter, a 5-fold lower dose equal to 14.8 mg/kg and a 2-fold higher dose equal to 148 mg/kg] were determined and a dose volume of 0.3 ml/mouse was assigned. Trastuzumab as a lyophilized commercial product, was first reconstituted in sterile water for injection according to the product label (440 mg/20 ml) and then further dilution in 0.9% sodium chloride (normal saline) was carried out. The control group was administered normal saline and each of the other 3 groups received one of the three concentrations of Trastuzumab (14.8, 74 or 148 mg/kg) by slow intravenous (IV) injection into the tail vein every 21 days for 84 days. The doses were prepared under sterile conditions. For serum, peripheral pooled blood from each group was collected in approximately 3 ml aliquot after 14 days from injection. Sera were prepared from all collected blood samples for tested groups by allowing the blood contained in tubes to clot for a minimum of 30 min while the tubes in a vertical position at room temperature following by centrifugation at 3000×g for 10 min. The supernatants were separately aliquoted and stored at − 40 °C until use [34].

### In vitro detection of anti-trastuzumab antibody (Ab) in serum samples

This was carried out using either Affinity Capture Elution (ACE) assay or MTT cytotoxicity assay against MCF-7 cell line.

ACE assay was used for detecting anti-trastuzumab Ab in all serum samples recovered from patients and tested animals.

### ACE assay procedures

ELISA plates (Corning Incorporated, 96 Well EIA/RIA Plates, corning, NY 14831, USA) were coated with therapeutic humanized monoclonal antibody (Tmab) at a concentration of 5 μg/ml in BupH carbonate–bicarbonate buffer by adding 100 μl per well and incubating overnight at 4 °C. Samples were diluted 1:10 in TBS. Aliquots (100 μl each) were acidified with 50 μl 300 mM acetic acid and incubated at room temperature for 5 min. Tmab coated plates were washed three times with TBST and 50 μl 1 M Tris pH 9.5 were added to each well. Acid-treated samples (100 μl aliquots) were added to the buffered coated wells and the plates were incubated overnight at 4 °C. The following day, plates were washed three times with TBST followed by elution of bound ADA by addition of 65 μl 300 mM acetic acid for 5 min at room temperature. New Plates were then loaded with 50 μl aliquots of 1 M Tris pH 9.5 buffer. Aliquots (50 μl each) of the acid eluate were transferred to the plates containing the buffer solution followed by incubation for 1 h at room temperature to allow binding of eluted ADA to the wells. Plates were then washed three times with TBST and blocked with casein buffer (1% in PBS) for 1 h at room temperature. After washing three times with TBST, 100 μl aliquots B-Tmab conjugate were added and incubated at room temperature for 1 h to allow binding to plate bound ADA. Plates were then washed three times with TBST and 100 μl aliquots SA-HRP solution were added to the wells and the plates were incubated for 30 min at room temperature. After that, the plates were washed three times with TBST and 100 μl aliquots of 3,3′,5,5′ tetramethylbenzidine (TMB) substrate (Ortho-Clinical Diagnostics Pencoed, UK) were added to the wells and incubated for 30 min at room temperature. Color development was stopped by addition of 100 μl aliquots of 2 M phosphoric acid, and the plates were read at 450 nm in a Biotek ELx800 plate reader [13].

### MTT cytotoxicity assay

This assay was conducted using MCF-7 cell line and used for detecting anti-trastuzumab Ab in one human serum sample that showed the highest anti-trastuzumab Ab titer in ACE assay. The cells were treated in triplicate with trastuzumab (Herceptin 440 mg/20 ml) over a period of 72 h to determine the resistance and sensitivity of the breast cancer cell line to the monoclonal antibodies [35–37]. Cells were seeded at a density of 5 × 10^3^ cells/ml in volumes of 100 μl medium into 96-well plates and incubated in CO_2_ incubator at 37 °C and 5% CO_2_ for 24 h. Aliquots (100 μl each) of the trastuzumab dilutions were added to the cells in triplicates at concentrations of 500, 250, 125, 62.5, 31.25, 15.6, 7.8, 3.9, 1.95, 0.975, 0.49 and 0.245 μg per well to determine the optimal concentration for the cytotoxicity assay. Following incubation of cells for 72 h in CO_2_ incubator at 37 °C and 5% CO_2_, a 50 μl aliquots of MTT solution were added to each well and the plates were re-incubated for further 4 h followed by adding a 50 μl aliquot of DMSO to each well. Absorbance was measured immediately in a plate reader at 570 nm. Cytotoxicity was recorded as a percentage of that obtained for the control untreated cells (100%) [35].

MTT cytotoxicity assay was also conducted with trastuzumab (Herceptin 440 mg/20 ml) at the concentration giving 30% cytotoxicity (250 μg/well) in presence of 3 dilutions of the serum sample preparation containing anti-trastuzumab antibodies [1:20, 1:100 and 1:200] in triplicate. Cells suspension (5 × 10^3^ cells/ml) was seeded at 100 μl aliquots into 96-well plates and incubated in CO_2_ incubator at 37 °C and 5% CO_2_ for 24 h. The neutralizing activity of anti-trastuzumab Ab was tested at 3 different regimens: (i) 1st regimen, equal volumes (100 μl each) of the drug and serum preparations were added simultaneously to MCF-7 cell line; (ii) 2nd regimen, serum sample preparations (50 μl aliquots) were added to MCF-7 cell line 1 h before addition of the drug (50 μl/well); (iii) 3rd regimen, both drug and serum sample preparations were mixed at equal volumes and left at room temperature for 1 h before the addition of their mixture to MCF-7 cell line at 100 μl per well. Following incubation of cells for 72 h in CO_2_ incubator at 37 °C and 5% CO2, a 50 μl aliquots of MTT solution were added to the wells and the plates were re-incubated for further 4 h followed by adding a 50 μl aliquot of DMSO to each well. Absorbance was measured immediately in a plate reader at 570 nm. Cytotoxicity was recorded as a percentage of that obtained for the control untreated cells (100%).

## Data Availability

The datasets used and/or analyzed during the current study are included in the manuscript. All data are available upon request through the corresponding author of the manuscript.

## Abbreviations

mAb: monoclonal antibody
HER2: human epidermal growth factor receptor-2
Ab: antibody
ACE assay: An Affinity Capture Elution assay
ADAs: anti-drug antibodies
ATAs: anti-therapeutic antibodies
IG: immunogenic
nADAs: neutralizing ADAs
NABs: neutralizing antibodies
PK: pharmacokinetics
PD: pharmacodynamics
SC/IV: subcutaneous/ intravenous
CDRs: complementarity determining regions
AEs: adverse events
IRRs: infusion related reactions
Tmab: Trastuzumab
IRB: The Institution Review Board
NCI: National Cancer Institute
ICH-GCP: International Council for Harmonisation- Good Clinical Practice
AED: animal equivalent dose
IV: intravenous
TBS: Tris-Buffered Saline
B-Tmab: Biotin-Trastuzumab Conjugate
SA-HRP: Streptavidin poly-HRP
TMB: tetramethylbenzidine
